# Real-Time Characterization Using in situ RHEED Transmission Mode and TEM for Investigation of the Growth Behaviour of Nanomaterials

**DOI:** 10.1038/s41598-018-19857-2

**Published:** 2018-01-26

**Authors:** Janghyun Jo, Youngbin Tchoe, Gyu-Chul Yi, Miyoung Kim

**Affiliations:** 1 Department of Materials Science and Engineering and Research Institute of Advanced Materials, Seoul National University, Seoul, 08826 Korea; 2 Department of Physics and Astronomy, Institute of Applied Physics and Research Institute of Advance Materials, Seoul National University, Seoul, 08826 Korea

## Abstract

A novel characterization technique using both *in situ* reflection high-energy electron diffraction (RHEED) transmission mode and transmission electron microscopy (TEM) has been developed to investigate the growth behaviour of semiconductor nanostructures. RHEED employed in transmission mode enables the acquisition of structural information during the growth of nanostructures such as nanorods. Such real-time observation allows the investigation of growth mechanisms of various nanomaterials that is not possible with conventional *ex situ* analytical methods. Additionally, real-time monitoring by RHEED transmission mode offers a complete range of information when coupled with TEM, providing structural and chemical information with excellent spatial resolution, leading to a better understanding of the growth behaviour of nanomaterials. Here, as a representative study using the combined technique, the nucleation and crystallization of InAs nanorods and the epitaxial growth of In_x_Ga_1−x_As(GaAs) shell layers on InAs nanorods are explored. The structural changes in the InAs nanorods at the early growth stage caused by the transition of the local growth conditions and the strain relaxation processes that occur during epitaxial coating of the shell layers are shown. This technique advances our understanding of the growth behaviour of various nanomaterials, which allows the realization of nanostructures with novel properties and their application in future electronics and optoelectronics.

## Introduction

Understanding the growth mechanisms of nanomaterials is one of the major goals in materials science and solid-state physics^1–4^. Such knowledge will enable the fabrication of electronic and optoelectronic nanodevices having specific physical properties^5–7^. To study the growth behaviour of thin films in real time, *in situ* characterization using reflection high-energy electron diffraction (RHEED) has been widely employed^8,9^. The real-time monitoring technique of RHEED in reflection mode provides information about the surface structure of thin films because of the high surface sensitivity of the technique^9^. This *in situ* technique can avoid the possible structural or phase changes of a sample that arise from exposure to the atmosphere or from retarded annealing during cool down after growth and overcomes other difficulties associated with observing various intermediate states during growth^10,11^, thereby providing new, accurate information on the structure and defects of materials. In contrast to reflection mode, RHEED transmission mode can provide structural information of nanomaterials through the strong interaction of transmitted electrons with the entire nanomaterial. This principle is similar to that of electron diffraction in transmission electron microscopy (TEM), thereby enabling the study of the structural properties of nanomaterials during growth. RHEED transmission mode has recently been employed to identify the crystal structure and epitaxial relationship of nanostructures and to verify the two-/three-dimensional (2-D/3-D) growth mode transitions during growth^12–17^. However, in-depth studies of the growth mechanisms of nanomaterials using RHEED transmission mode have rarely been reported despite the great importance and potential of this technique. Acquiring structural information on nanomaterials in real time during their growth by RHEED transmission mode allows us to investigate their fundamental growth mechanisms in detail throughout the entire growth process, from nucleation to the fully grown nanostructures. Moreover, a complete range of structural analysis can be performed when RHEED transmission mode is coupled with TEM measurements, which exhibit excellent spatial resolution. The combined method provides an unprecedented scope and detailed knowledge of nanostructure growth. This includes various intermediate states, such as the nucleation of nanomaterials, structural changes and defect formation in nanostructures during growth, and annealing in a growth chamber.

Here, we report a novel real-time characterization method using *in situ* RHEED transmission mode and TEM, particularly to demonstrate the feasibility of the method for studying the growth behaviour of nanostructures and to unravel the growth mechanisms of InAs nanorods in detail, which distinguishes this study from previous works that showed only basic knowledge of nanostructure growth. Such a combined technique allowed us to examine details of the growth behaviour of InAs nanorods and In_x_Ga_1−x_As(GaAs) coaxial nanorods that could not be obtained by traditional *ex situ* analytical methods. Remarkably, we proved that the catalyst-free growth of InAs nanorods was initiated and that nanorods with uncommon thick layers of zincblende (ZB) or wurtzite (WZ) phases formed at the very initial growth stage, and these layers were later transformed into the typical heavily twinned structures. This result is in contrast with the common belief that only stacking faults and twin defects energetically prefer to form on nanorods under typical As-rich growth conditions^18–20^. Furthermore, we clearly revealed how the lattice constants evolved and how strain relaxation occurred during the early stages of coaxial coating of GaAs and In_x_Ga_1−x_As shell layers. These results could provide a basis for the fabrication of stacking fault-free III-V compound semiconductor nanorods or for controlling the internal strain induced by lattice mismatch at the heterointerfaces of coaxial nanorods, which are essential for achieving the desired electronic and optoelectronic properties of their quantum heterostructures. Moreover, such real-time observations have significant implications for facilitating the development of new growth procedures through the valuable feedback provided by the structural information obtained by *in situ* RHEED transmission mode during growth.

## Results and Discussion

### Principles of RHEED transmission mode and its advantages over other techniques

**Figure 1:**
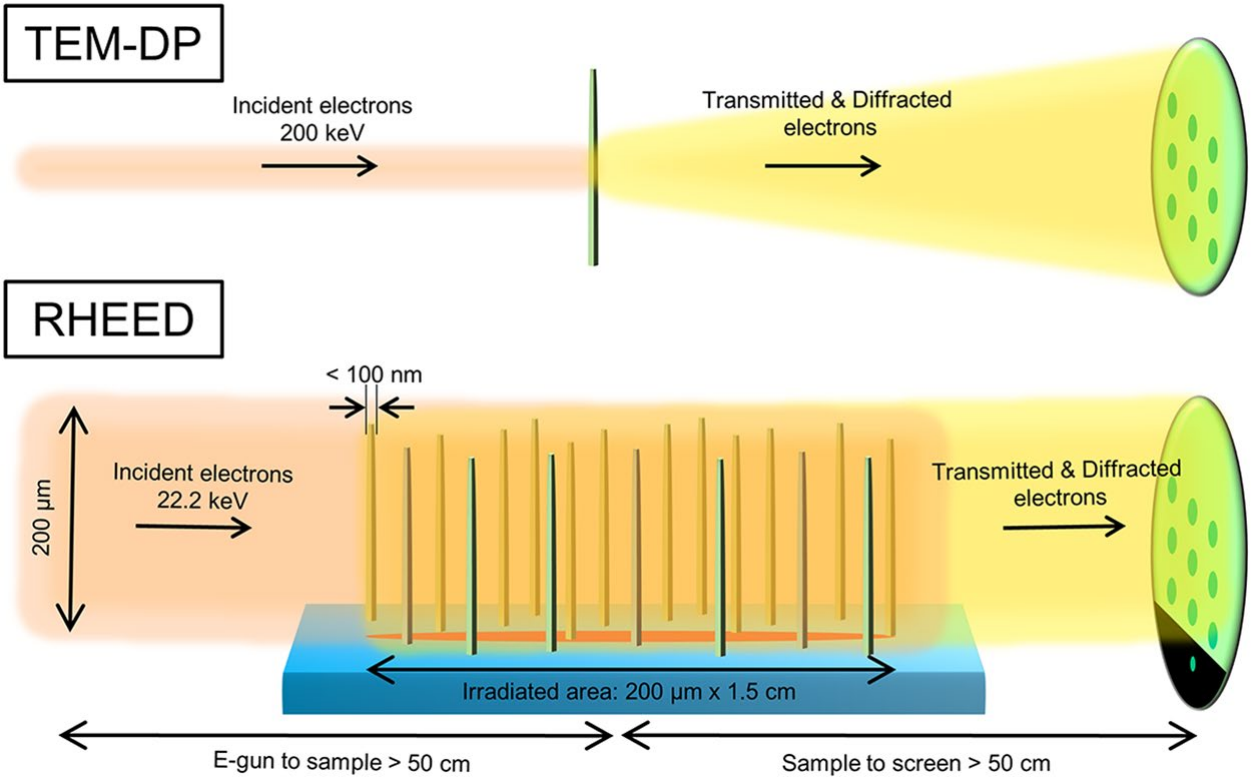
Schematic illustration of electron diffraction in TEM and RHEED. The RHEED electron beam has a spot size of 200 μm and glances off the surface of the substrate at an angle of *ca*. 0.8°. The size of the area irradiated by the electron beam is *ca*. 200 μm × 1.5 cm. The large spot size indicates that the RHEED diffraction pattern provides information concerning the nanostructures over a wide area.

The principles of RHEED transmission mode are schematically compared with those of electron diffraction in TEM in Fig. 1. A high-energy collimated electron beam from the RHEED gun passes through and interacts with the nanostructures, *i.e*., nanorods and nanotubes, that stand vertically on a substrate. Electrons with an energy of a few tens of keV from the RHEED gun are strong enough to transmit through nanorods having a diameter less than 100 nm. The volume (or surface area) fraction of nanorods having a density of 5 × 10^8^ cm^−2^ is less than 5%, indicating that there is sufficient free space for electrons to travel to the detector and produce diffraction patterns without significant transmittance loss. These diffraction spots appear in the positions in which the Bragg condition is satisfied, analogous to the typical diffraction methods such as X-ray diffraction or electron diffraction in TEM. This method can be easily employed with existing growth methods, *i.e*., pulsed laser deposition or molecular beam epitaxy (MBE), without modifying either the equipment or the special gun alignment. This flexibility generally allows for the application of RHEED transmission mode to the *in situ* structural analysis of nanostructures during growth.

RHEED transmission mode provides a more complete range of information when combined with TEM. This technique is fundamentally similar to electron diffraction in TEM but offers complementary information to that obtained with TEM, thereby allowing us to exploit the advantages of both techniques, *i.e*., real-time observation and structural analysis during growth by RHEED transmission mode and in-depth structural and chemical analyses with excellent spatial resolution by TEM. This real-time characterization technique also allows us to obtain structural information on nanostructures without retarding annealing during cool down after growth or the conventional sample preparation processes, which causes structural changes in the sample and thus restricts the analysis of the nucleation and growth mechanisms. Additionally, RHEED transmission mode allows structural information to be obtained for the entire nanorod because of the large electron beam size, thus providing representative characteristics of the whole sample, which are not achieved in local measurements by TEM. Therefore, using the combined techniques improves the understanding of the growth mechanisms of any kind of nanostructure from the atomic scale to the microscale, which is not attainable using traditional *ex situ* analytical methods or RHEED alone.

### Feasibility of RHEED transmission mode for structural analysis of nanorods

The growth behaviour of InAs nanorods on Si(111) was investigated using our real-time characterization technique. This material system was especially favourable for characterization because the nanorod diameter was sufficiently small for electrons to transmit through them, and their crystal orientations were well-aligned both in-plane and out-of-plane on the Si(111) substrate. Additionally, their various structural properties, which include the crystal phases and stacking faults that depend on the growth conditions, the nanorod size and the controllable composition of ternary compounds^20–23^, provide numerous aspects that can be explored using the *in situ* observation technique.

InAs nanorods were epitaxially grown on the Si(111) substrate by catalyst-free MBE. The high-density InAs nanorods were vertically well-aligned on the substrate. The nanorods normally showed a diameter and height of 40–60 nm and 2–3 μm, respectively. The coaxial coating of In_x_Ga_1−x_As and GaAs layers on the InAs core nanorods increased the diameter of the nanorods up to *ca*. 100 nm (Supplementary Information, Fig. S1). The homogeneous growth of the nanorods was confirmed within 2 inches of the Si substrate in an MBE chamber by scanning electron microscopy and TEM measurements of many nanorods, which is much larger than the size of electron beam focused on the substrate.

**Figure 2:**
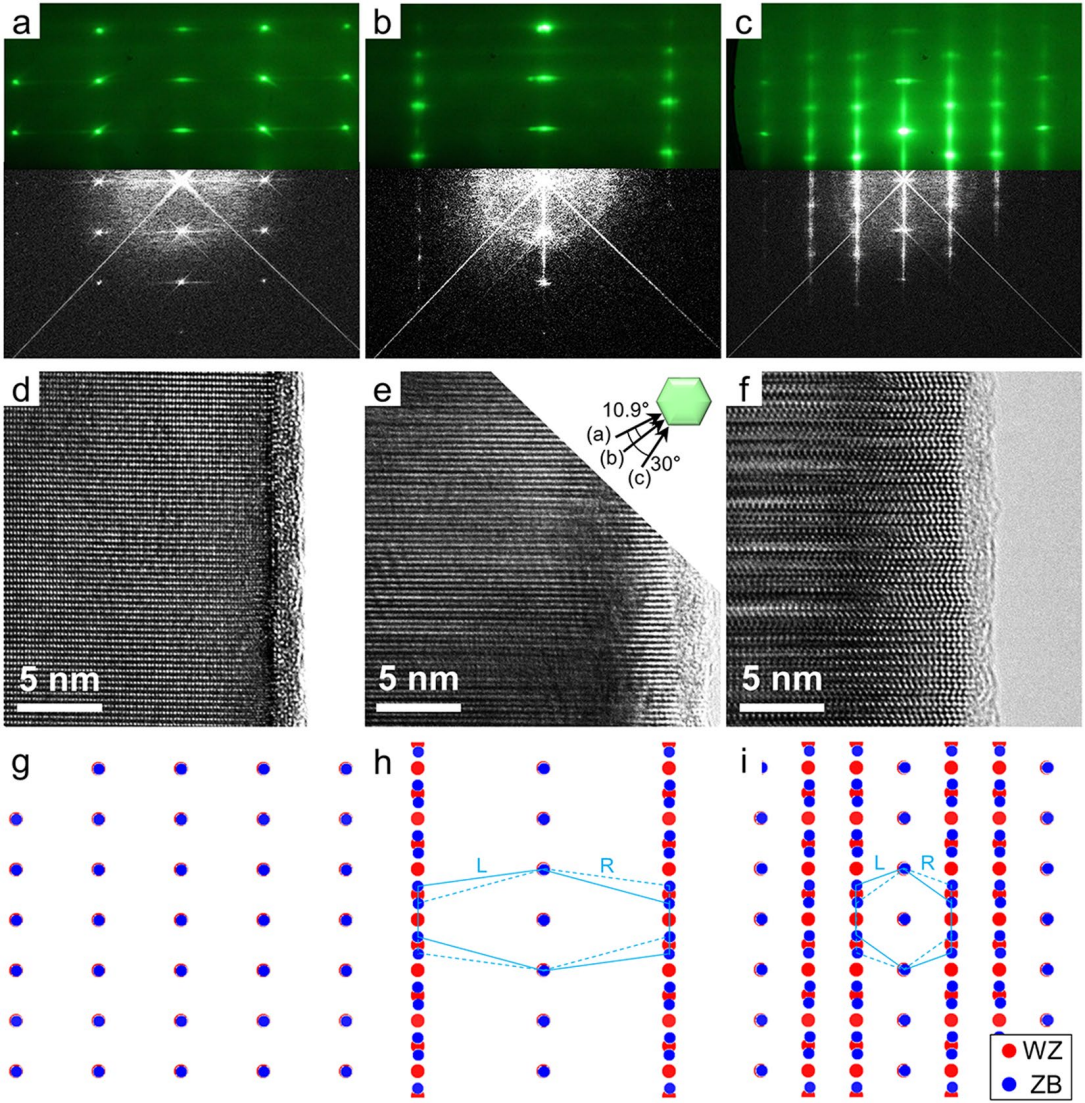
Correlation between the RHEED and TEM diffraction patterns. (**a**–**c**) Overlap of (top) the RHEED patterns and (bottom) the corresponding FFT patterns obtained from the HR-TEM images in (**d**–**f**) taken along the directions of (**a**) ${[10\overline{1}0]}_{WZ}/{[2\overline{1}\overline{1}]}_{ZB}$, (**b**) ${[41\overline{5}0]}_{WZ}/{[3\overline{2}\overline{1}]}_{ZB}$ and (**c**) ${[11\overline{2}0]}_{WZ}/{[1\overline{1}0]}_{ZB}$, respectively. (**d**–**f**) HR-TEM images of the InAs nanorods taken along the same three orientations. The inset in (**e**) displays the relationship between these orientations. (**g**–**i**) Simulated electron diffraction patterns along the same three orientations as (**a**–**c**), respectively. The red and blue dots represent the diffraction spots of WZ and ZB, respectively. The patterns in (**h**) and (**i**) also consist of symmetric twinned ZB structures.

RHEED patterns were recorded along three different orientations in the growth chamber after nanorod growth was completed (Fig. 2a–c, top). Figure 2a shows the single crystalline RHEED pattern obtained along the twinning direction of ${[10\overline{1}0]}_{WZ}/{[2\overline{1}\overline{1}]}_{ZB}$, where twin defects cannot be observed, while Fig. 2c shows a streaky pattern obtained along the ${[11\overline{2}0]}_{WZ}/{[1\overline{1}0]}_{ZB}$ direction, which is commonly used for twin observation. Such twin defects are normally created by the 60° rotation of the three In-As bonds of a tetrahedral unit around the growth direction at the topmost surface layer of the InAs nanorods during the incorporation of adatoms, which leads to the formation of stacking faults in the nanorods^24^. The RHEED pattern in Fig. 2b was acquired along the ${[41\overline{5}0]}_{WZ}/{[3\overline{2}\overline{1}]}_{ZB}$ direction between the orientations presented in Fig. 2a and c, as displayed in the inset of Fig. 2e.

The feasibility of applying RHEED transmission mode to the structural analysis of nanorods was verified by directly comparing the result with those from electron diffraction in TEM. High-resolution TEM (HR-TEM) images were taken at the edge of the InAs nanorods along the same three orientations as in the RHEED diffraction geometry (Fig. 2d–f), and fast Fourier transform (FFT) patterns were obtained from these HR-TEM images (Fig. 2a–c, bottom). Figure 2d and a shows a single crystalline lattice image of a nanorod and the corresponding spotty FFT pattern, respectively, whereas Fig. 2f and c shows a high density of the rotational twin in the same nanorod and the resulting streaky FFT pattern along the growth direction^23,25,26^. The scale of the RHEED pattern was adjusted to that of the FFT micrograph from the HR-TEM images by using the Si peaks as a reference; the distances between the RHEED streaks originating from the Si(111) substrate were compared with those between the FFT peaks from Si. The comparison of the two diffraction patterns in Fig. 2a–c revealed that the RHEED patterns perfectly matched the FFT patterns for all orientations, indicating a direct correlation between RHEED transmission mode and electron diffraction in TEM.

Additionally, the simulated diffraction patterns obtained from the twinned ZB and WZ phases of InAs confirmed that the RHEED patterns were truly identical to the diffraction patterns from TEM. The two simulated diffraction patterns were overlapped around their origins (Fig. 2g–i). The twinned ZB diffraction patterns are also presented as having L or R chirality in the images (details concerning the diffraction patterns of each phase can be found in Fig. S2 of the Supplementary Information). Comparison of the RHEED and simulated diffraction patterns revealed that the RHEED patterns also coincided with the mixed simulation patterns for all of the orientations; the experimental RHEED and FFT patterns showed additional streaks originating from the high density of stacking faults on the nanorods, which was not considered in the electron diffraction simulations. This agreement strongly suggested that the spotty RHEED patterns indeed reproduced the electron diffraction patterns of the 3-D materials in TEM, which is not the case when RHEED is used in the conventional reflection mode. These results indicate that RHEED transmission mode offers the same structural information about nanostructures that electron diffraction in TEM does. Such RHEED patterns can also be monitored in real time during the growth of nanostructures, as mentioned in the previous section. This fact demonstrates the utility of using RHEED transmission mode together with TEM for *in situ* structural observations and analyses of nanostructures.

Only the RHEED patterns of the vertically well-aligned InAs nanorods are shown; however, the RHEED patterns obtained from nanorods with poor alignments and epitaxial relationships also provide a range of information, such as the crystal growth direction, degree of vertical alignment and in-plane misorientation of the entire nanorod (see details in Fig. S3 of the Supplementary Information)^14,27^. In the present study, we will consider only the epitaxially grown nanorods with good vertical alignment, which are advantageous for diverse applications, including electronic and optoelectronic devices^28^.

### Initial growth behaviour of InAs nanorods

**Figure 3:**
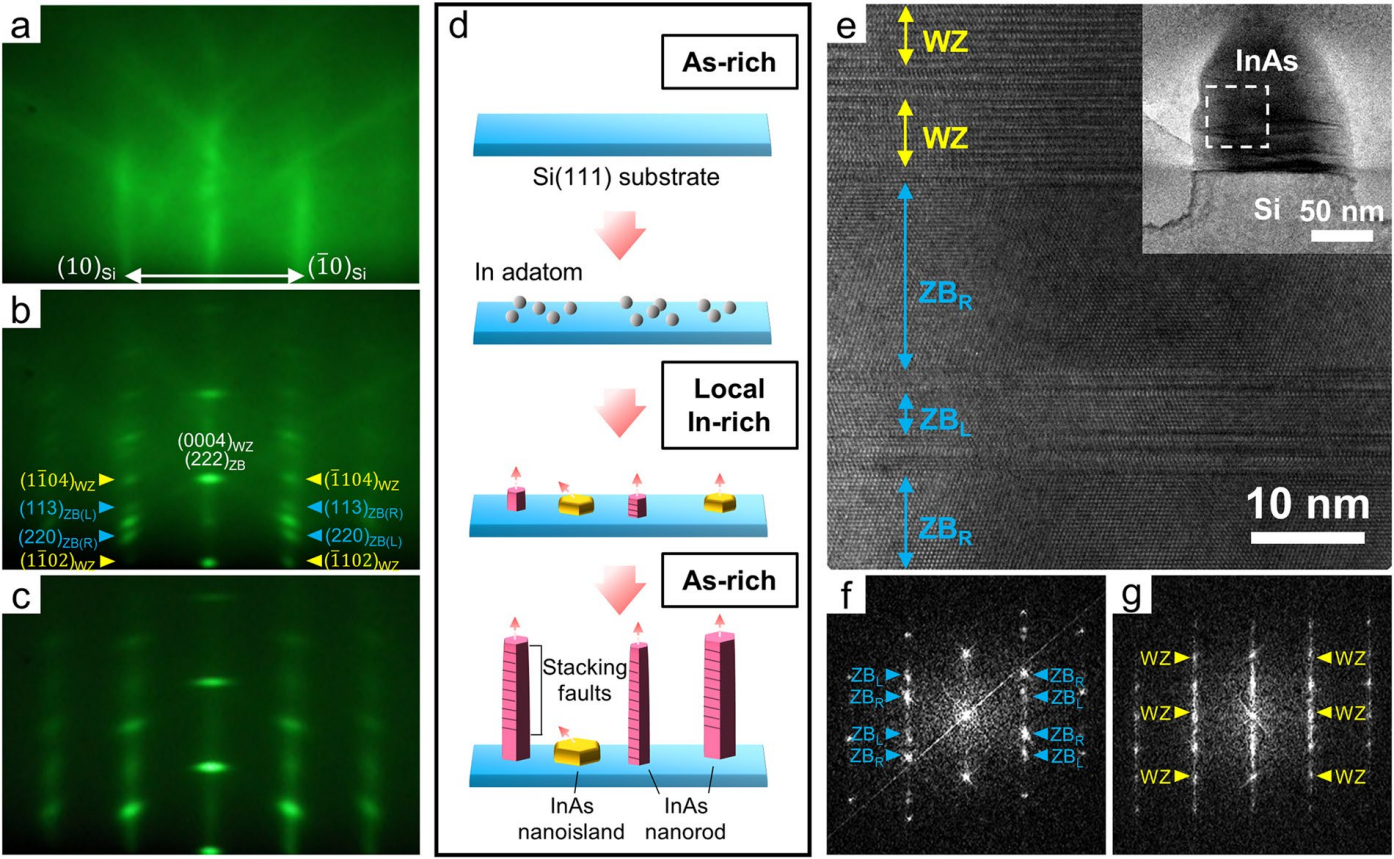
Real-time observation of the initial growth behaviour of InAs nanorods on the Si(111) substrate. (**a**–**c**) RHEED patterns obtained (**a**) just before the start of growth and (**b**) 30 s and (**c**) 15 min after growth commencement. The streaks in (**a**) and the diffraction spots in (**b**) are indexed in the figures. (**d**) Schematic illustration of the growth behaviour of the InAs nanorods on the Si(111) substrate. (**e**) HR-TEM image of an InAs nanorod grown for 30 s taken at the region indicated in the inset. The inset shows a low-magnification image of the InAs nanorod. (**f**,**g**) FFT patterns obtained for the (**f**) twinned ZB and (**g**) WZ regions in (**e**). Twin-ZB and WZ-sensitive spots are denoted by blue and yellow arrows, respectively, in (**f**) and (**g**). All of the RHEED and TEM diffraction patterns were taken along the ${[11\overline{2}0]}_{WZ}/{[1\overline{1}0]}_{ZB}$ direction to distinguish the ZB and WZ spots.

Next, we proceeded to investigate the initial growth behaviour of InAs nanorods on oxide-etched Si(111) using RHEED transmission mode coupled with TEM measurements. Figure 3a–c shows the RHEED patterns that were acquired at different growth stages. The image in Fig. 3a was taken before growth began; the streaks were derived from the surface of the Si(111) substrate. A spotty pattern emerged, and the streaks from the surface of the Si(111) substrate disappeared a few seconds after growth began, as shown in Fig. 3b (Supplementary Information, Fig. S4). Such a sudden appearance of Bragg spots at the very initial stage suggested the catalyst-free crystal growth of InAs nanorods on the Si(111) substrate, in contrast to the gradual appearance of a spotty RHEED pattern caused by self-catalysed VLS growth^16^. These Bragg spots appeared at different locations to those of the streaks from the Si substrate, which indicated an abrupt change in the d-spacing and, thus, the instantaneous in-plane strain relaxation of the InAs nanorods. The instantaneous disappearance of the streaky patterns also indicated that the RHEED patterns obtained during growth did not originate from the substrate but rather from the InAs nanorods. Additionally, the observed RHEED pattern showed epitaxial relationships of InAs ${[10\overline{1}0]}_{WZ}/{[2\overline{1}\overline{1}]}_{ZB}||{\rm{Si}}\,[2\overline{1}\overline{1}]$ and InAs(0002)_*WZ*_/(111)_*ZB*_||Si(111).

It is notable that the characteristic spots, the so-called twin-ZB and WZ-sensitive spots^29^, appeared during the early growth stage of the InAs nanorods (Fig. 3b) and nearly disappeared at a growth time of 15 min (Fig. 3c). The twin-ZB spot was a superimposed double-diffraction pattern from the twinned ZB phase in an individual nanorod that had both L and R chirality or from two types of nanorods that had either L or R chirality, as indicated by the blue arrows in Fig. 3b. The WZ-sensitive spots are a periodic array of ${[1\overline{1}02]}_{WZ}$ reflections, as indicated by the yellow arrows in Fig. 3b. The unexpected appearance of these characteristic peaks and their subsequent disappearance were attributed to the transformation of the growth behaviour during InAs nanorod growth. At growth initiation, the InAs nanorods might be composed of relatively thick ZB and WZ layers rather than the commonly reported heavily twinned structures, as evidenced by the observation of their characteristic peaks in the diffraction pattern (Fig. 3b). As the growth proceeded, InAs nanorods with a typical disordered structure and a high density of rotational twins began to predominantly grow, which resulted in the streaky features and lack of distinct intensity of the characteristic spots in the diffraction pattern; such a heavily disordered layer stacking could no longer be indexed to ZB and WZ^26,29,30^. This pattern exactly corresponded to the streaky RHEED pattern shown in Fig. 3c that was obtained from almost fully grown nanorods.

The transformation of the growth behaviour of the InAs nanorods was proven using subsequent TEM measurements. InAs nanorods were grown for only 30 s under the same growth conditions, and cross-sections of the nanorods were prepared by focused ion beam (FIB) milling. The cross-sectional HR-TEM image in Fig. 3e reveals that most of the nanorods in the early growth stage had relatively thick ZB and WZ layers with more than 10–50 atomic layers, whereas fully grown nanorods normally exhibited a periodicity of only a few atomic layers. The FFT patterns (Fig. 3f and g) obtained from these ZB and WZ layers contained clearly defined twin-ZB and WZ-sensitive spots, as indicated by the blue and yellow arrows, respectively, which were responsible for the superimposed characteristic spots found in the RHEED pattern in Fig. 3b. Such relatively thick ZB and WZ layers remained at the base of the fully grown InAs nanorods (see Fig. S5 in the Supplementary Information). These experimental findings are in good agreement with the observations from *in situ* RHEED, which indicated a transition in the growth behaviour at the initial growth stage of the InAs nanorods.

The growth behaviour of the InAs nanorods was closely associated with the transition of the growth conditions from In-rich to As-rich. Under the high As overpressure used in the present study, the energetically most favoured As-rich (2 × 2) reconstructed (111)B surface structure appeared, whose unit cell consists of As trimers^18–20^. This growth condition modified the surface energies and kinetics for the preferential stacking faults and rotational twin defect formation on the (111)B growth plane^18–20^. However, this was not the case in the earliest stage of InAs nanorod growth. The large amount of In species required for the nucleation of nanorods under high As-rich growth conditions arrive at specific sites, such as the edge of oxides remaining on the Si substrate or Si nanocrystals^31,32^. In adatoms could be easily supplied to the nucleation sites by their high diffusivity on the Si substrate at a very early stage. Such a local In-rich condition resulted in a distinct surface structure other than the As-rich (2 × 2) reconstructed (111)B surface^20,33^, which possibly allowed the formation of relatively thick ZB and WZ layers, even in the As-rich growth conditions^34,35^. The local In-rich condition on the substrate in the early growth stage was also evidenced by the fast growth rate of nanorods (200 nm/85 s = 8 μm/h) because of the high supply of In adatoms, compared with the overall growth rates of nanorods (3–4 μm/80 min = 2–3 μm/h)^26,33^. The As-rich conditions were then immediately restored, while the supply rate of In species to the tip of an individual nanorod decreased because of the increasing diameter and density of the InAs nanorods. This eventually resulted in the formation of rotational twins in the nanorods (Fig. 3d).

A point to be further considered when analysing RHEED patterns is that they provide structural information concerning the nanorods over a wide area. Various types of InAs from nuclei to fully grown nanorods coexist on a substrate during growth because nucleation continues to occur simultaneously with the growth of nanorods. The RHEED patterns obtained during the growth of InAs nanorods reveal averaged signals that include newly created nuclei as well as growing nanorods. Such averaged information obtained from the nanorods at different stages of their lives might make it difficult to examine the structural information of nanorods at a specific growth stage; thus, we need to be very cautious when interpreting RHEED patterns. Nevertheless, we can thoroughly investigate the growth behaviour of nanorods both at the very initial stage and at the end of growth. For the very early growth stage, only nuclei exist over the whole surface of the Si substrate, which generates RHEED patterns showing the structural information of nuclei alone, as shown in Fig. 3b. On the other hand, the RHEED intensity is mainly influenced by the fully grown nanorods at the end of growth. Nucleation minimally occurs within the small regions occupied by growing nanorods because most of nearby adatoms are already consumed. Thus, the nucleation rate decreases as growth proceeds. This results in small amount of nuclei compared to growing nanorods, which means that the RHEED pattern mainly arises from fully grown long nanorods, as shown in Fig. 3c. (Supplementary Information, Fig. S6) Such characteristics of the nanorod growth allow us to extract the structural information of nanorods both at the nucleation stage and at the end of growth, which are of interest in the present study, despite the averaging effect of RHEED transmission mode.

### Strain relaxation of the In_x_Ga_1−x_As and GaAs shell layers during epitaxial growth

**Figure 4:**
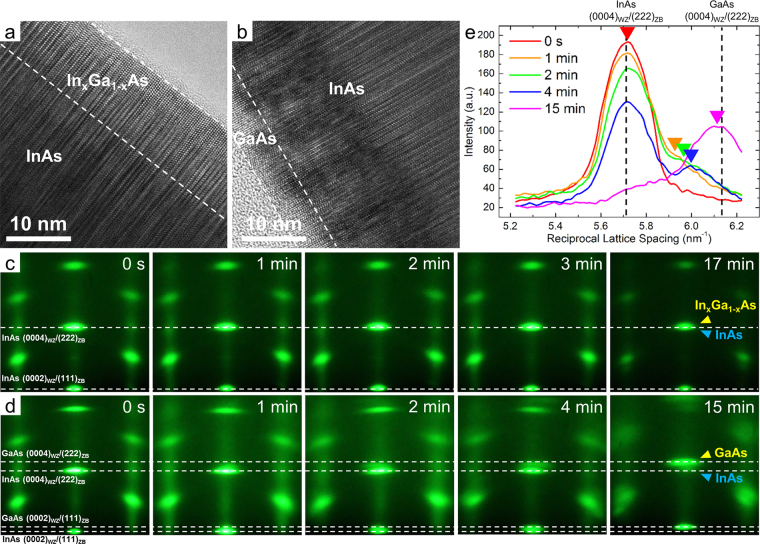
Real-time observation of the coaxial coating of InAs core nanorods. (**a**,**b**) HR-TEM images of (**a**) In_x_Ga_1−x_As/InAs and (**b**) GaAs/InAs coaxial nanorods. (**c**,**d**) Series of RHEED patterns taken during (**c**) In_x_Ga_1−x_As and (**d**) GaAs coating of InAs nanorods. All of the RHEED patterns are aligned with respect to their transmitted beams as zero points, which are not shown in the images. The growth time for each RHEED pattern is indicated in the image. All of the diffraction patterns were obtained along the ${[11\overline{2}0]}_{WZ}/{[1\overline{1}0]}_{ZB}$ direction. (**e**) RHEED intensity profiles around the InAs(0004)_*WZ*_/(222)_*ZB*_ and GaAs(0004)_*WZ*_/(222)_*ZB*_ spots in (**d**) at growth times of 0, 1, 2, 4 and 15 min. The RHEED intensities were measured along the central vertical line intersecting the (000 *l*)_*WZ*_ diffraction spots. The positions of the GaAs peaks at which the GaAs spots appeared are indicated by arrows on the plots. The expected positions of the GaAs and InAs spots corresponding to their lattice constants of (0002)_*WZ*_/(111)_*ZB*_ and (0004)_*WZ*_/(222)_*ZB*_ are indicated by dashed lines in (**c**–**e**).

We further investigated the growth behaviour of In_x_Ga_1−x_As and GaAs shell layers on InAs nanorods in real time using RHEED transmission mode. Figure 4a and b shows HR-TEM images obtained along the ${[11\overline{2}0]}_{WZ}/{[1\overline{1}0]}_{ZB}$ direction at the edges of In_x_Ga_1−x_As/InAs (0.8 < x < 0.85) and GaAs/InAs coaxial nanorods, respectively. The interfaces and edges of the coaxial nanorods were marked with white dashed lines by comparing the HR-TEM images with the energy dispersive X-ray spectroscopy (EDS) line scan results (details concerning the thickness and chemical compositions of the coaxial nanorods are provided in Fig. S7 of the Supplementary Information). The RHEED patterns were acquired at different growth stages during the growth of the In_x_Ga_1−x_As and GaAs coating layers (Fig. 4c and d). The first images in Fig. 4c and d reveal the spotty and streaky patterns from the InAs core nanorods, which are identical to the pattern shown in Fig. 3c. The coaxial coating of the In_x_Ga_1−x_As shell layers did not cause significant or observable changes in the RHEED diffraction patterns (Fig. 4c). This result strongly suggests that the In_x_Ga_1−x_As coating layers, which had almost no lattice mismatch with InAs, grew epitaxially, with almost the same crystal structures and lattice constants as the InAs core nanorods. The only difference was that the intensities of the RHEED spots and streaks decreased as growth proceeded because of the increased thickness of the coaxial nanorods, which decreased the transmission probability of electrons through the nanocrystals.

In contrast to the In_x_Ga_1−x_As shell layers, the positions of the RHEED diffraction spots shifted during the coaxial coating of GaAs. This shift was because of the relatively larger lattice mismatch of *ca*. 7% between GaAs and InAs. The RHEED spots from GaAs split off from those of InAs during the initial stage of coaxial coating and gradually evolved to positions matching the lattice constants of GaAs. This evolution is evident in Fig. 4d for the GaAs(0002)_*WZ*_/(111)_*ZB*_ and (0004)_*WZ*_/(222)_*ZB*_ spots. Additionally, the intensities of the GaAs and InAs spots changed as growth proceeded: those for InAs darkened, while those for GaAs brightened. Such intensity changes were attributed to an increase in the amount of GaAs shell layers and to the relatively low transmittance of the RHEED electron beam through the thick InAs core nanorods compared with that through the thin GaAs shell layers.

To trace the evolution of the RHEED diffraction spots in more detail, the intensity profiles around the InAs(0004)_*WZ*_/(222)_*ZB*_ and GaAs(0004)_*WZ*_/(222)_*ZB*_ spots were examined at the different growth stages (Fig. 4e). The graph shows that the GaAs spots abruptly appeared and gradually shifted over time to locations corresponding to the GaAs lattice constants. The transitional behaviour of the RHEED spots suggested that the GaAs coaxial coating layers formed epitaxially on the InAs core nanorods with the same crystal structure and lattice constants during the early growth stage and then reverted to the GaAs lattice constants as growth proceeded.

**Figure 5:**
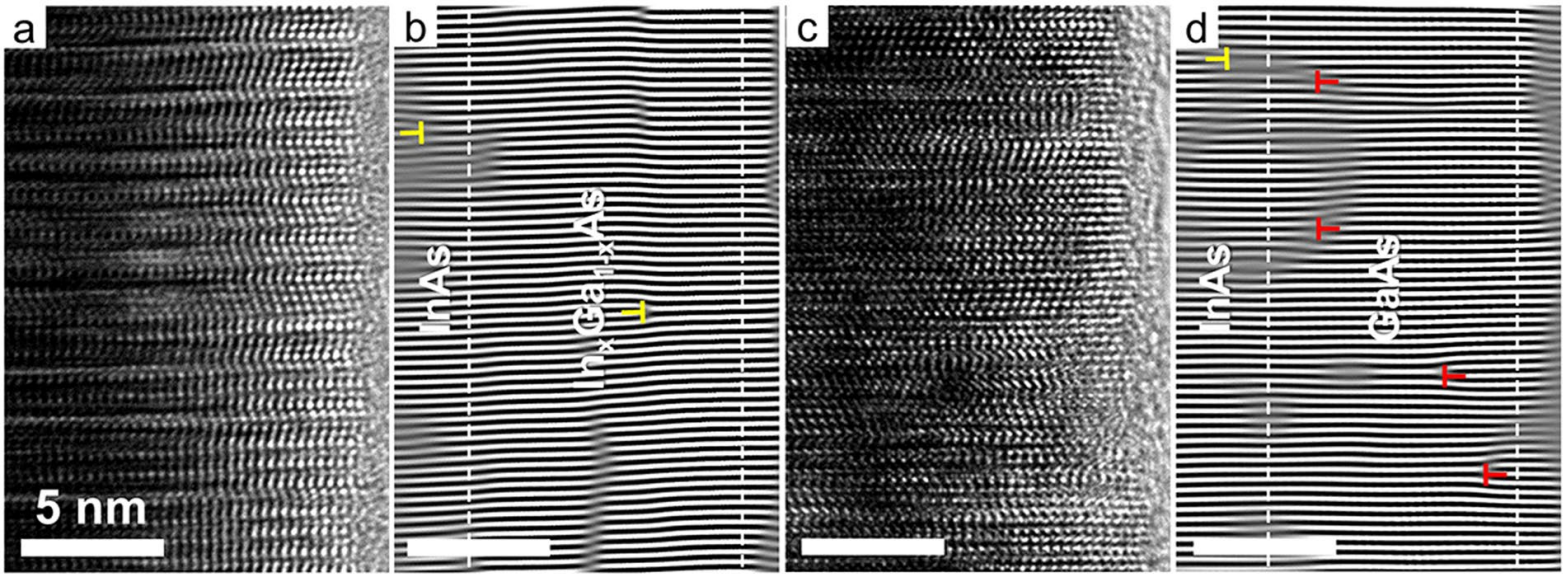
Microstructural defects at the interface of the coaxial nanorods. **(a,c)** HR-TEM images of the In_x_Ga_1−x_As/InAs and GaAs/InAs coaxial nanorods obtained along the ${[11\overline{2}0]}_{WZ}/{[1\overline{1}0]}_{ZB}$ direction. (**b**,**d**) Corresponding Fourier-filtered images obtained by selecting the FFT (0002)_*WZ*_/(111)_*ZB*_ spots of (**a**) and (**c**), respectively. The dislocation locations are indicated by the ‘T’ symbol. The yellow and red colours of the symbol represent extra half-planes inserted towards the InAs core nanorod and towards the edge of the shell layer, respectively. The edge of the nanorods and the interfaces between the core and shell layers were identified by EDS line scans and are indicated by the white dashed lines in (**b**) and (**d**).

We also analysed the Fourier-filtered HR-TEM images of the In_x_Ga_1−x_As and GaAs coaxial nanorods to relate their microstructural characteristics to the movement of the RHEED spots. Figure 5b and d displays the Fourier-filtered images obtained from the corresponding HR-TEM images of In_x_Ga_1−x_As and GaAs coaxial nanorods shown in Fig. 5a and c, respectively. While the In_x_Ga_1−x_As shell layers showed almost no dislocations near the interface, the GaAs shell layers commonly showed dislocations originating from the large lattice mismatch between GaAs and InAs. The dislocations were not observed at the highly mismatched GaAs/InAs interface but rather were mostly observed within the GaAs shell layers. The generation of extra half-planes corresponding to (0002)_*WZ*_/(111)_*ZB*_ in the GaAs shell layers resulted from strain relaxation during their growth, which was responsible for the gradual evolution of the GaAs RHEED spots and lattice constants observed in Fig. 4d and e. Such a delayed strain relaxation in the shell layers might be attributed to the lack of suitable glide systems for the motion of the misfit dislocations or to the relaxation kinetics impeding dislocation generation and movement, such as the barrier to dislocation nucleation and the interaction between dislocations and other defects^36–40^. Notably, this commensurate growth behaviour of the nanorods with large curvature followed by the relaxation processes at the nanometre scale is analogous to that of conventional epitaxial growth of thin films on a substrate with different lattice constants.

## Conclusion

In conclusion, we demonstrated that RHEED transmission mode coupled with TEM measurements can be successfully used for real-time observation and comprehensive study of the detailed growth mechanism of InAs nanorods and In_x_Ga_1−x_As(GaAs) coaxial nanorods. RHEED transmission mode provides real-time information that complements the information from TEM imaging with excellent spatial resolution. This combined technique revealed the intermediate states of catalyst-free growth and the structural changes in the InAs nanorods during the very initial growth period, revealing the local transition of the growth conditions from In-rich to As-rich. Furthermore, we observed the real-time evolution of the lattice constants during the epitaxial coating of GaAs shell layers, which indicated a gradual strain relaxation process by the introduction of dislocations. It is difficult to obtain these new findings by conventional *ex situ* analytical methods alone. Additionally, these results are expected to be valuable for controlling the growth of functional nanostructures. Optimum growth conditions for the fabrication of desirable nanostructures can be explored through the feedback provided by real-time observation using the RHEED transmission mode; the In/As flux ratio and growth temperature can be controlled and optimized through repeated experiments, for example, for the fabrication of stacking fault-free III-V nanorods or strain-controlled coaxial nanorods. This provides the opportunity to realize their novel properties and applications in future electronics and optoelectronics. More importantly, we believe that this work will stimulate a new field of research concerning the *in situ* observation of nanostructure growth and has great potential to enhance our understanding of crystal growth and other areas where time-resolved analysis is required for in-depth investigation of phenomena.

## Methods

### MBE growth of InAs nanorods and In_x_Ga_1−x_As(GaAs)/InAs coaxial nanorods

InAs nanorods and In_x_Ga_1−x_As(GaAs)/InAs coaxial nanorods were grown by catalyst-free molecular beam epitaxy (MBE) on an oxide-etched Si(111) substrate (Supplementary Information, Fig. S1). An oxide-free Si surface was prepared by etching with buffered oxide etch for 30 s and cleaning in deionized water for 30 s followed by annealing at 650 °C for 10 min with As_4_ introduced inside an ultrahigh vacuum preparation chamber. The InAs nanorods were grown at 500 °C for 80 min in a cryogenically cooled ultrahigh vacuum MBE growth chamber (RIBER 32P) using high-purity In and uncracked As_4_ molecular beams from Knudsen cells. As_4_ was supplied to the Si(111) substrate 10 min before supplying In to prevent In droplet formation on the substrate. To obtain high-quality and well-controlled In_x_Ga_1−x_As(GaAs)/InAs coaxial nanorods, a two-step growth procedure was followed. InAs nanorods were grown at 500 °C for 36 min, and In_x_Ga_1−x_As shell layers were subsequently grown on the InAs core nanorods at 400 °C for 40 min using an additional Ga molecular beam. GaAs/InAs coaxial nanorods were also prepared as a control group by coating GaAs shell layers on the InAs core nanorods at 400 °C for 15 min. To produce uniform shell coating layers on the InAs core nanorod, the substrate was rotated approximately twice per second.

### *In situ* RHEED characterization

The entire growth process was monitored in real time using RHEED without special alignment of the RHEED gun (electron beam energy: 22.2 keV; spot size: 0.2 mm). The length of the electron beam irradiated along the beam trajectory was 15 mm, indicating an incident angle of the electron beam of *ca*. 0.8° with respect to the substrate surface. Real-time videos of the RHEED diffraction patterns were acquired using a commercial digital single-lens reflex camera having a time resolution of 0.04 s. The lattice parameters of the InAs nanorods and In_x_Ga_1−x_As(GaAs)/InAs coaxial nanorods were estimated by comparing the spacings between the RHEED spots with those of the Si(111) substrate as a reference. The growth of the InAs core nanorods was not interrupted while taking snapshots and recording videos of the RHEED patterns. The snapshots of the RHEED patterns presented here were acquired with a longer exposure time of 0.5 s to obtain high quality images. In contrast, RHEED patterns were taken while the growth of coaxial shell layers was temporarily ceased for 30 s–1 min to obtain RHEED patterns along the specific crystallographic orientations of the nanorods because the substrate was rotated to obtain uniform shell coating layers. The rotation of the substrate was stopped and the In and Ga shutters were closed during the measurements. The As source was still introduced into the growth chamber while taking snapshots to prevent decomposition of the coaxial nanorods.

### TEM sample preparation and TEM measurements of the nanorods

Structural analysis of the InAs nanorods and In_x_Ga_1−x_As(GaAs)/InAs coaxial nanorods was performed by TEM. For TEM sample preparation, the nanorods were dispersed in anhydrous ethanol and dropped onto a holey carbon-coated TEM grid. The cross-sections of InAs nanorods grown for only 30 s were prepared by FIB milling in an FEI-Helios 650 FIB instrument. Bright-field and HR-TEM images along with the corresponding FFT data were obtained with a 200-kV field-emission TEM instrument (JEOL, JEM-2100F).

### Data Availability

The datasets generated during and/or analysed during the current study are available from the corresponding author on reasonable request.

## Electronic supplementary material

Supplementary Information
