# Supplementary material for: Real-Time Characterization Using in situ RHEED Transmission Mode and TEM for Investigation of the Growth Behaviour of Nanomaterials
Source: Sci Rep. 2018 Jan 26;8:1694. doi: 10.1038/s41598-018-19857-2 (PMC5786047; doi:10.1038/s41598-018-19857-2)
Supplement: Supplementary file 1 — Supplementary Information [file 41598_2018_19857_MOESM1_ESM.doc]

**Supplementary Information**

**Real-Time Characterization Using in situ RHEED Transmission Mode and TEM for Investigation of the Growth Behaviour of Nanomaterials**

Janghyun Jo^1^, Youngbin Tchoe^2^, Gyu-Chul Yi^2,*^ and Miyoung Kim^1,*^

^1^Department of Materials Science and Engineering and Research Institute of Advanced Materials, Seoul National University, Seoul 08826, Korea

* E-mail: mkim@snu.ac.kr

^2^Department of Physics and Astronomy, Institute of Applied Physics and Research Institute of Advance Materials, Seoul National University, Seoul 08826, Korea

* E-mail: gcyi@snu.ac.kr


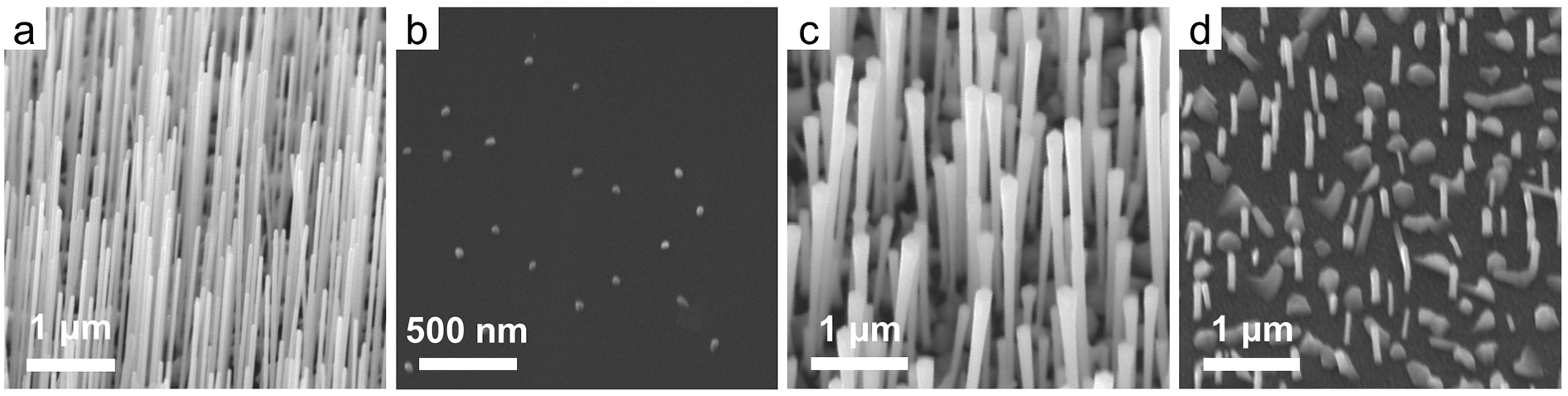


**Figure S1.** Scanning electron microscope (SEM) images showing the surface morphology of nanorods grown on Si(111). **(a)** InAs nanorods. **(b)** InAs nanorods grown only for 30 s. **(c)** In_x_Ga_1-x_As/InAs coaxial nanorods. **(d)** GaAs/InAs coaxial nanorods.

InAs nanorods and coaxial nanorods of In_x_Ga_1-x_As/InAs and GaAs/InAs were grown on Si(111) substrate. InAs nanorods and In_x_Ga_1-x_As/InAs coaxial nanorods extended up to a few micrometres in length show excellent vertical alignment, as shown in Fig. S1a and c, even though they were grown under the exposure of electron beam during the whole growth process to obtain RHEED patterns. They were not bent or damaged by the irradiation of electron beam, indicating that electron beam hardly influences on the growth of nanorods. This might be due to low current density of large-area electron beam irradiation. The growth of InAs nanorods for GaAs coating was carried out only for a short time (*ca.* 2.5 min) to prevent possible bending of the long GaAs/InAs coaxial nanorods because of the large lattice mismatch between GaAs and InAs, and high stiffness of GaAs shell layer compared with InAs core nanorod (Fig. 1Sd).


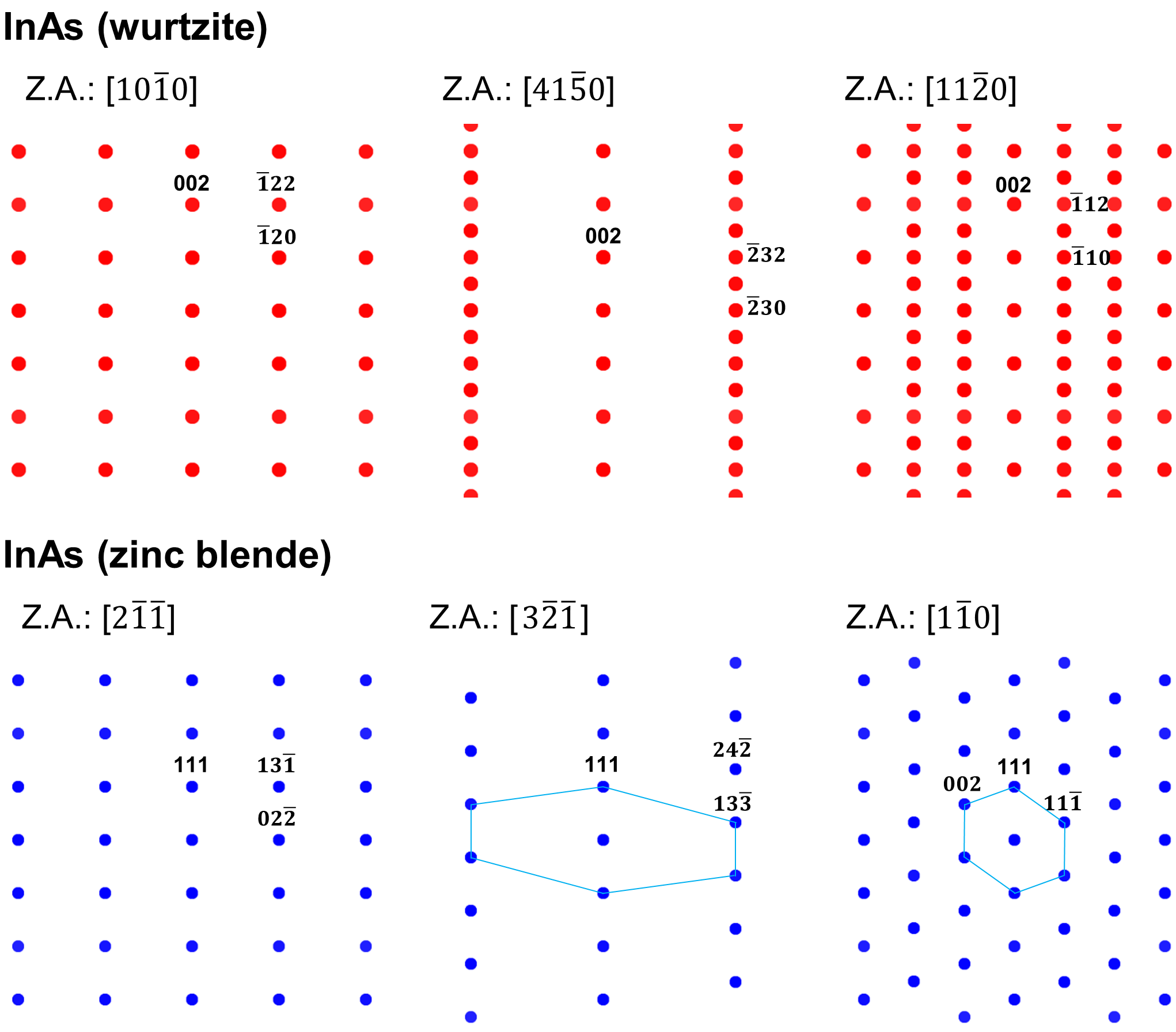


**Figure S2.** Simulated electron diffraction patterns of InAs WZ and ZB phases obtained along the three different orientations. The diffraction patterns in Fig. 2 were produced by overlapping those of WZ and ZB at each orientation. Twinned ZB diffraction patterns in Fig. 2h and i were obtained by twinning corresponding ZB patterns on the (111) plane and overlapping them around their origins. The typical intermixed structure observed for the InAs nanorods could readily occur because of the very small energy difference between the ZB and WZ polytypes, *i.e.*, *ca.* 5 meV/atom,^1^ as well as their atomically identical (0001) and $\{111\}$ facets.


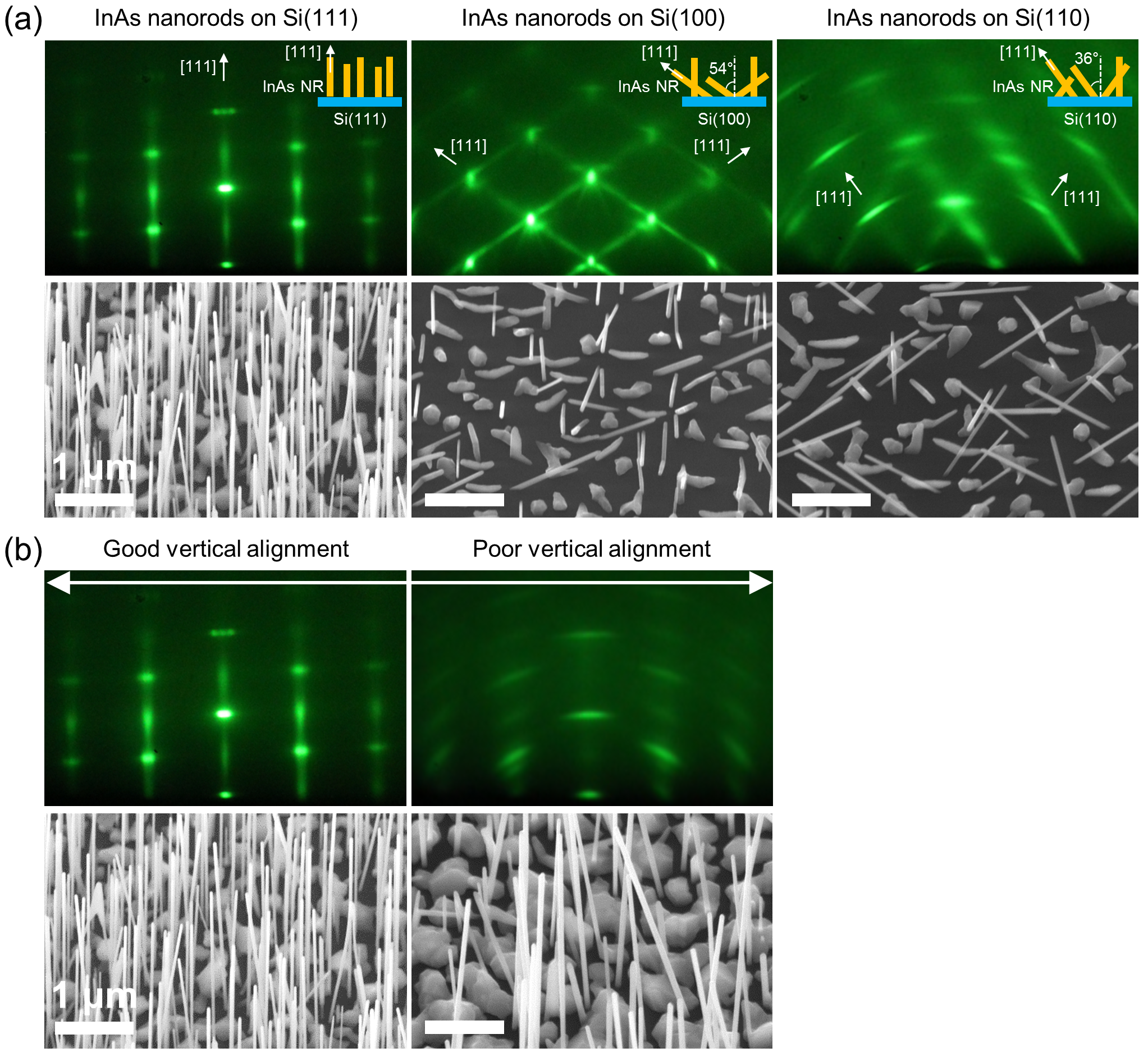


**Figure S3.** RHEED patterns of poorly-aligned InAs nanorods. **(a)** (top) RHEED patterns of InAs nanorods grown on Si(111), Si(100) and Si(110) substrates. (bottom) Corresponding SEM images of InAs nanorods. A schematic diagram of InAs nanorods representing their crystal growth direction is displayed in each RHEED pattern. **(b)** (top) RHEED patterns depending on the vertical alignment of InAs nanorods grown on Si(111) substrates. (bottom) Corresponding SEM images of InAs nanorods. All of the RHEED patterns were acquired along the $[11\overline{2}0]_{WZ}$/$[1\overline{1}0]_{ZB}$ direction of the nanorod.

Figure S3a shows various RHEED patterns depending on the crystal growth direction of the InAs nanorods. The different growth directions of the nanorods were obtained by using different surface orientations of Si substrates.^2^ The nanorods were epitaxially grown in a direction normal to the (111) plane of the Si substrate, vertically on the Si(111) substrate or tilted 54° and 36° away from the surface normal on Si(100) and Si(110), respectively, as shown in the schematic diagrams and SEM images in Fig. S3a. The RHEED patterns constructively generated from these nanorods reflect their structural information, *i.e.*, diffraction spots of (111) point towards the direction in which the nanorods were grown. This indicates that the dominant growth direction of an entire nanorod can be easily obtained by analysing the RHEED patterns.

The degree of vertical alignment of the nanorods can be also predicted from the RHEED patterns.^3^ Figure S3b shows the RHEED patterns depending on the vertical alignment of the InAs nanorods grown on Si(111) substrates. InAs nanorods were sometimes bent during growth due to inappropriate growth conditions, as shown in the right SEM image in Fig. S3b. Such bent nanorods were responsible for dispersed RHEED patterns, whereas vertically well-aligned nanorods produced clear spotty patterns. The spread of the RHEED patterns increased as the degree of vertical alignment of the nanorods decreased, as shown in the RHEED patterns in Fig. S3b.


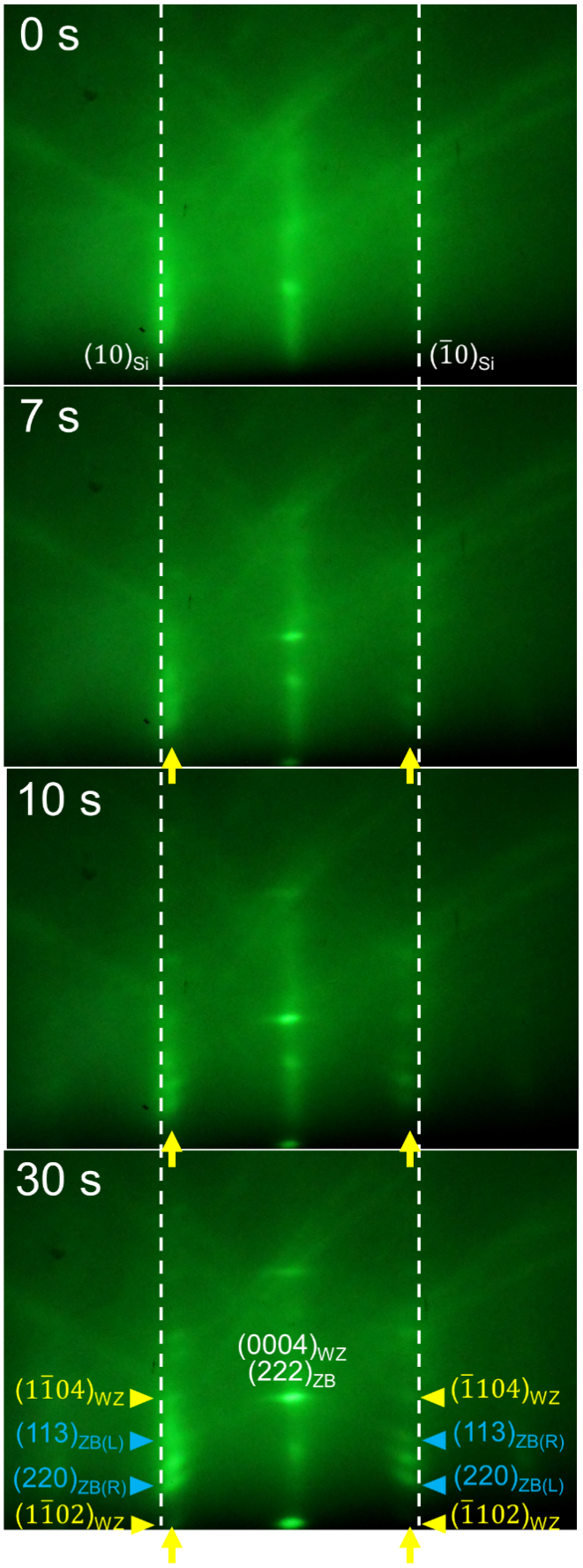


**Figure S4.** Series of RHEED patterns taken at the very initial growth stage of the InAs nanorods. All of the RHEED patterns are aligned with respect to their transmitted beams as zero points, which are not shown in the images. The growth time of the nanorods for each RHEED pattern is indicated in the image. All of the RHEED patterns were obtained along the $[11\overline{2}0]_{WZ}$/$[1\overline{1}0]_{ZB}$ direction. The positions of the streaks from the Si substrate and the spotty patterns from the InAs nanorods are indicated by white dashed lines and yellow arrows, respectively.

Figure S4 clearly shows that the spotty patterns from the InAs nanorods appear a few seconds after growth began. These spotty patterns are positioned at locations different from those of the streaks from the Si substrate from their first appearance in the RHEED pattern. We used the RHEED pattern at 30 s in the manuscript because the intensity of the InAs spotty pattern is high enough to differentiate the characteristic spots of the ZB and WZ phases, which are identified with blue and yellow arrows, respectively, in Fig. S4.


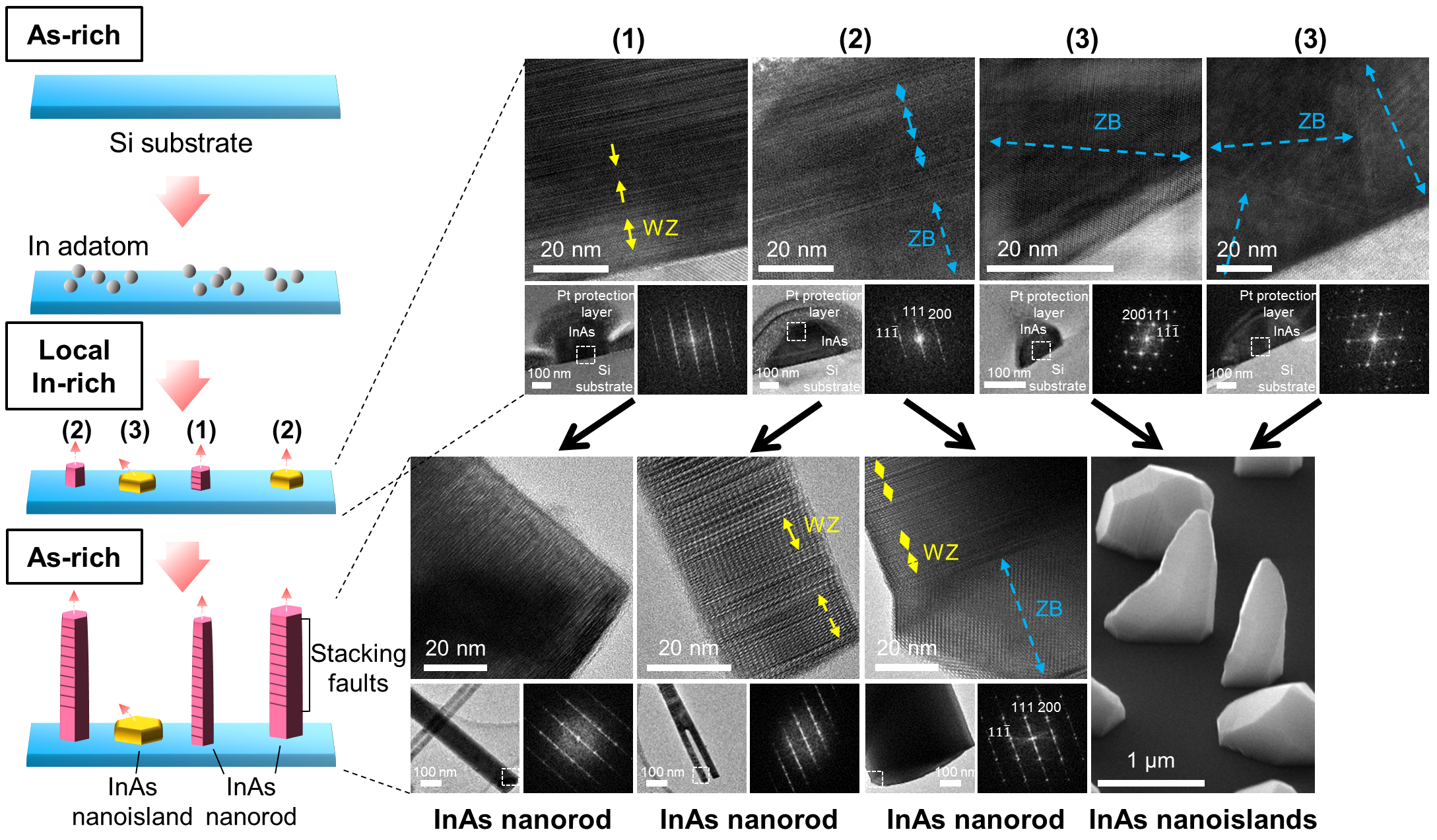


**Figure S5.** Crystal structures at the base of InAs nanorods. The four image sets at the top show the crystal structures of the InAs nanorods at the interface with the Si(111) substrate at an early stage of growth. The four lower image sets show the crystal structures of fully-grown InAs nanorods and nanoislands. Each image set consists of a low-magnification BF image of InAs nanorods, HR-TEM image obtained from the region indicated by the dashed square in the BF image and FFT micrograph obtained from the HR image. The last image at the bottom is SEM image showing InAs nanoislands. ZB and WZ layers free of stacking faults are indicated in the figures by dashed arrows along the [0001]_WZ_/[111]_ZB_ growth direction. The data shown here are representative of more than 10 nanorods.

The appearance of streaks in the FFT micrographs indicates that the InAs nanorods are composed of heavily twinned structures with alternating WZ and ZB phases. As shown in case (1), a high density of stacking faults formed even at the very early stages of growth, which led to the formation of nanorods without thick ZB and WZ layers. Thick ZB and WZ layers, however, developed for most of the nanorods at the initial stage, which still remained at the base of the fully-grown nanorods (case (2)). Nanoislands, which did not grow into nanorods with high aspect ratio, normally formed when the initial growth proceeded in a growth direction other than [0001]_WZ_/[111]_ZB_ (case (3)). These nanoislands, however, rarely contribute to the formation of RHEED patterns because the number of nanoislands is very small compared to that of nanorods and their crystallographic orientations are not aligned each other well enough to give constructive interference pattern. Even though the density of nanoislands is higher than normal, in particular, at non-optimized growth conditions, the random crystallographic orientations of nanoislands prevent the formation of their RHEED patterns as shown in Fig. S3.


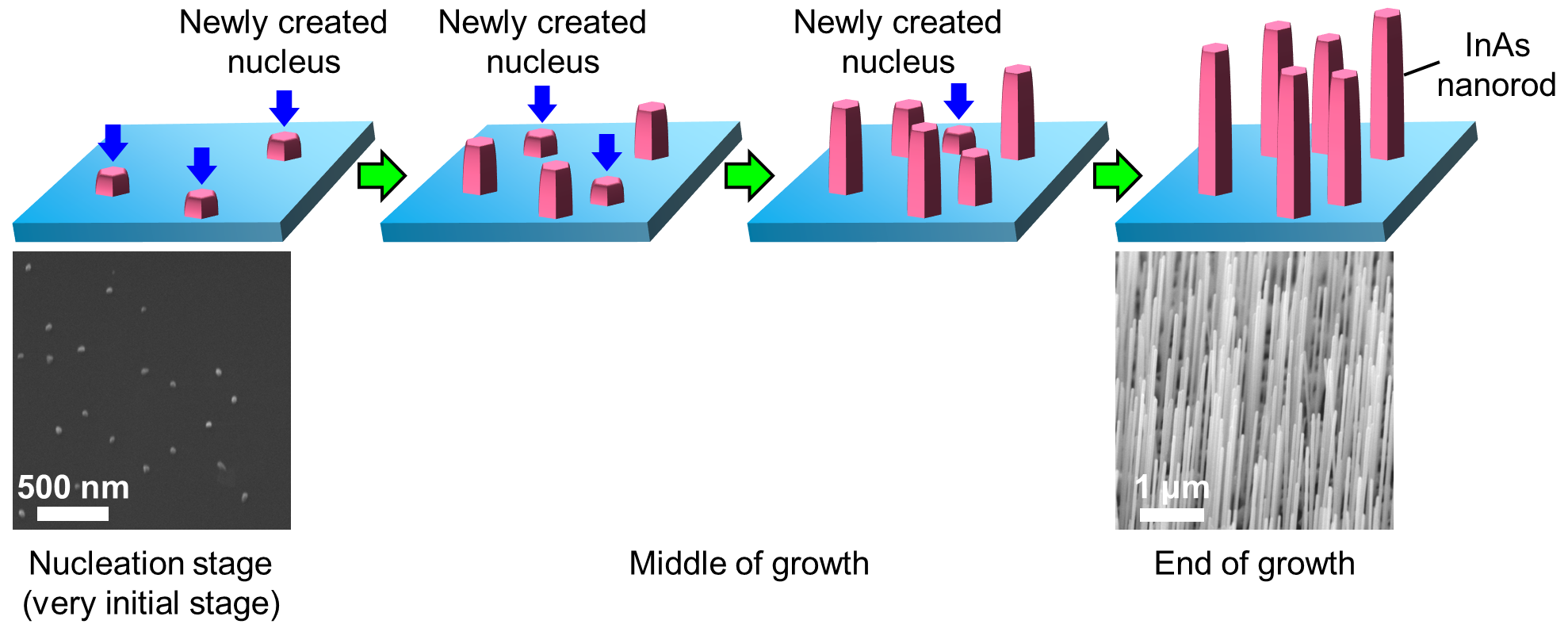


**Figure S6.** Schematic illustrations showing the growth behaviour of the InAs nanorods at each stage. Nuclei form on the substrate at the very initial stage of growth and grow into nanorods. Nucleation continues to occur simultaneously with the growth of nanorods; thus, various types of InAs from nuclei to growing nanorods coexist in the middle of the growth period. Nucleation minimally occurs within the small regions occupied by the growing nanorods since most of nearby adatoms are already consumed by the growing nanorods; thus, the nucleation rate decreases as growth proceeds. At the end of growth, fully grown long nanorods mainly exist on the substrate. The corresponding SEM images, which show only nuclei at the nucleation stage and fully grown nanorods at the end of growth, are presented in the figure.


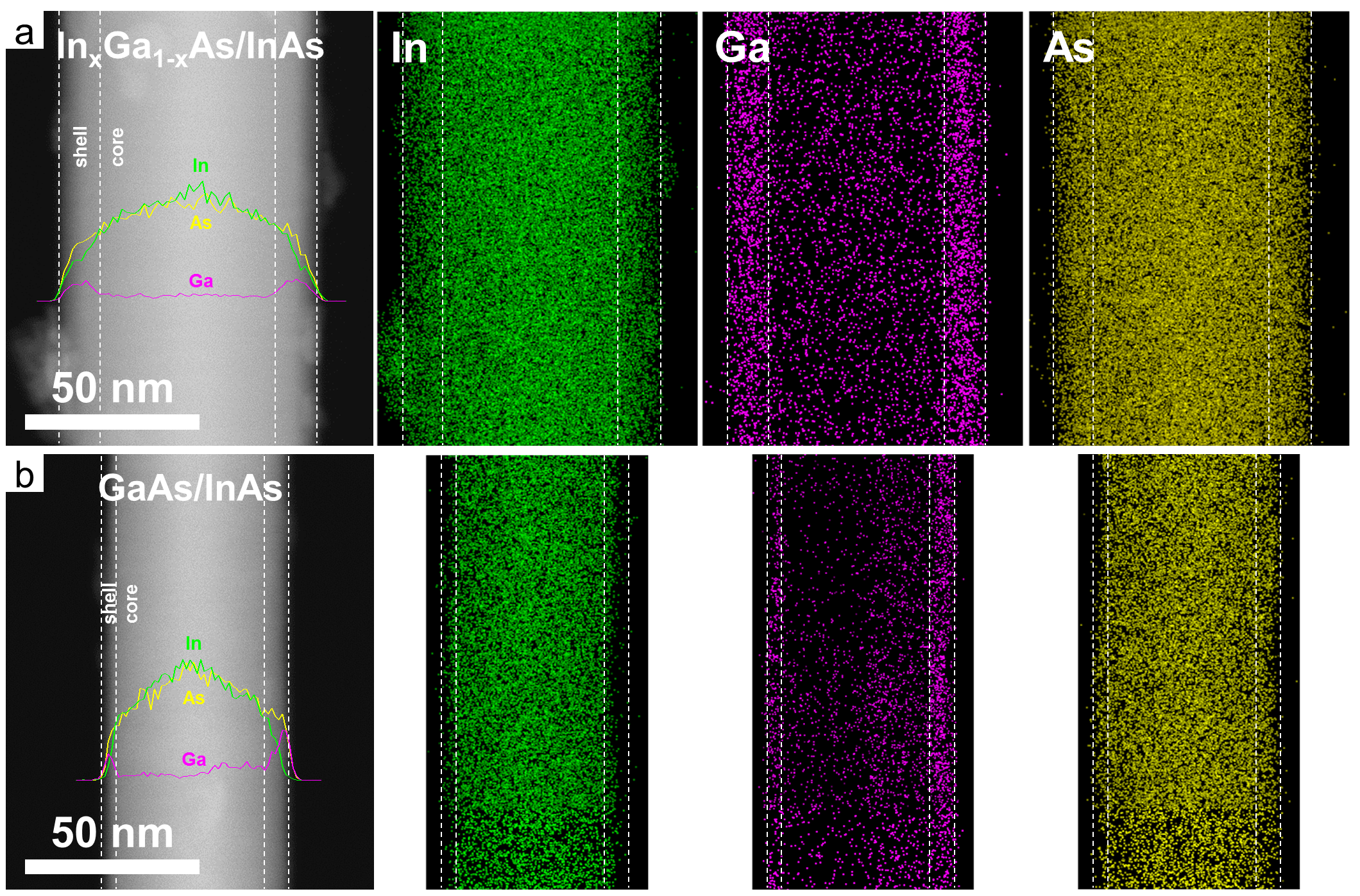


**Figure S7.** In_x_Ga_1-x_As/InAs and GaAs/InAs coaxial nanorods. High-angle annular dark-field scanning TEM (HAADF-STEM) images of **(a)** In_x_Ga_1-x_As/InAs and **(b)** GaAs/InAs coaxial nanorods and corresponding EDS element maps of In, Ga and As, respectively. EDS line profiles of In, Ga and As were obtained across the coaxial nanorods and displayed in each HAADF-STEM image. The interfaces and edges of the coaxial nanorods were marked by white-dashed lines by comparing HAADF-STEM images and EDS element maps and line profiles.

EDS line profiles and elemental maps in Fig. S7 reveal that In_x_Ga_1-x_As and GaAs shell layers were grown well on InAs core nanorods. In_x_Ga_1-x_As layers grown for 40 min were measured to be 12-nm-thick whereas GaAs layers grown for 15 min were 6-min-thick. We can guess the thickness of shell layers depending on the growth time since the growth rate of shell layers is well investigated in our MBE chamber through previous numerous experiments; for example, GaAs shell layers corresponding to RHEED patterns in Fig. 4d are expected to have thicknesses of 4 Å for 1 min, 8 Å for 2 min, 16 Å for 4 min, and 6 nm for 15 min.

**REFERENCES**

1 Akiyama, T., Sano, K., Nakamura, K. & Ito, T. An empirical potential approach to wurtzite-zinc-blende polytypism in group III-V semiconductor nanowires. *Jpn. J. Appl. Phys.* **45,** L275-L278 (2006).

2 Soshnikov, I. P., Cirlin, G. E., Tonkikh, A. A., Nevedomskii, V. N., Samsonenko, Y. B. & Ustinov, V. M. Electron diffraction on GaAs nanowhiskers grown on Si(100) and Si(111) substrates by molecular-beam epitaxy. *Phys. Solid State* **49,** 1440-1445 (2007).

3 Songmuang, R., Landre, O. & Daudin, B. From nucleation to growth of catalyst-free GaN nanowires on thin AlN buffer layer. *Appl. Phys. Lett.* **91,** 251902 (2007).
